# Multiple Novel Ceftazidime-Avibactam-Resistant Variants of *bla*_KPC-2_-Positive Klebsiella pneumoniae in Two Patients

**DOI:** 10.1128/spectrum.01714-21

**Published:** 2022-05-19

**Authors:** Qingyu Shi, Renru Han, Yan Guo, Yang Yang, Shi Wu, Li Ding, Rong Zhang, Dandan Yin, Fupin Hu

**Affiliations:** a Institute of Antibiotics, Huashan Hospital, Fudan University, Shanghai, China; b Key Laboratory of Clinical Pharmacology of Antibiotics, Ministry of Health, Shanghai, China; c Department of Clinical Laboratory Medicine, Second Affiliated Hospital of Zhejiang University, Hangzhou, China; Pennsylvania State University

**Keywords:** carbapenem-resistant *Enterobacteriaceae*, ceftazidime-avibactam, *bla*_KPC-33_, *bla*_KPC-35_, *bla*_KPC-76_, *bla*_KPC-78_, *bla*_KPC-79_, ST11, ST859

## Abstract

As the first-line antimicrobial agent for the infection caused by carbapenem-resistant *Enterobacterales*, ceftazidime-avibactam develops drug resistance during its ever-growing clinical use. In this study, we report multiple novel variants in *bla*_KPC-2_-positive Klebsiella pneumoniae from two separate patients during their exposure to ceftazidime-avibactam. For one patient, the *bla*_KPC-2_ gene carried by K. pneumoniae mutated into *bla*_KPC-35_, *bla*_KPC-78_, and *bla*_KPC-33_ over the same period, while that for the other patient mutated into *bla*_KPC-79_ and further evolved into *bla*_KPC-76_ to enhance resistance level, among which *bla*_KPC-76_ and *bla*_KPC-79_ were reported for the first time. In contrast with *bla*_KPC-2_, the emergent mutations within the Ω-loop conferred high-level resistance to ceftazidime-avibactam with a sharp reduction of carbapenemase activity. These *bla*_KPC_-positive K. pneumoniae isolated from sputum (both patients) and cerebrospinal fluid (patient 2) belonged to ST11 and ST859, respectively. All strains located *bla*_KPC_ alleles on IncFII/IncR plasmids, except one on an IncFII plasmid. Such *bla*_KPC-2_ variants first appeared after 9 to 18 days of ceftazidime-avibactam usage, but the lack of its feasible detection method often led to the assumption of ceftazidime-avibactam sensitivity resulting in clinical incorrect usage. Subsequent substitution of ceftazidime-avibactam with carbapenems also failed, because the *bla*_KPC-2_-containing K. pneumoniae dominated again. Ultimately, treatment failed even with the therapeutic regimen of ceftazidime-avibactam combined with carbapenems, because of the inadequate concentration of avibactam in infection sites and decreased drug sensitivity of strains caused by increased expression of *bla*_KPC_ and point mutation of *ompK35* and *ompK36*. As novel KPC variants conferring resistance to ceftazidime-avibactam are constantly emerging worldwide, quick and efficient laboratory detection and surveillance are urgently needed for infection control.

**IMPORTANCE** Carbapenem-resistant K. pneumoniae which was classified as the most urgent threat by World Health Organization, is the most critical public health concern due to its high mortality rate. Recently, the rapid mutation of *bla*_KPC_ has occurred during anti-infective therapy, which posed an unexpected challenge for both the diagnostic laboratory and clinical practice.

## INTRODUCTION

Carbapenem-resistant *Enterobacterales* (CRE) with extensive-drug resistance have been increasingly reported worldwide, which was usually accompanied with high mortality rates, because the available therapeutic regimens for their infection treatments were limited (1). Klebsiella pneumoniae carbapenemase-producing Klebsiella pneumoniae (KPC-KP) occupied the most proportion in CRE. It was almost entirely *bla*_KPC-2_ in China but *bla*_KPC-3_ and *bla*_KPC-2_ (with a ratio of about 3:1) in some American and European countries (2–5). Currently, tigecycline, colistin, and ceftazidime-avibactam, described as the “three swordsmen,” are the best available therapeutic options for the infections caused by CRE (6). For the effective inhibition of serine-carbapenemase (KPC and OXA-48-like), ceftazidime-avibactam presents an excellent *in vitro* activity and safety profile, and was then endorsed as the first-line drug for the treatment of CRE-related infections in China in 2019 (7, 8).

Although ceftazidime-avibactam initially demonstrated potent activity against *Enterobacterales* in China (the susceptibility rate of K. pneumoniae could reach 93.8%), the resistance has gradually emerged with clinical usage (5). Besides the production of metallo-β-lactamase, the main resistance mechanism of K. pneumoniae to ceftazidime-avibactam was the high expression of *bla*_KPC-2_ gene combined with OmpK35 porin deficiency and OmpK36 porin mutation before the drug was approved for clinic use (9–11). However, following the wide clinical application of ceftazidime-avibactam, KPC-KP acquiring resistance through a single mutation in *bla*_KPC-2_ or *bla*_KPC-3_ gene gradually appeared worldwide, with mutations mostly occurring in the Ω-loop (Arg164 to Asp179), the Val240-loop (Cys238 to Thr243) between β3- and β4-strand, and the Lys270-loop (Ala267 to Ser275) between the β5-strand and the α11-helix (12, 13). In China, new *bla*_KPC_ subtypes including *bla*_KPC-33_, *bla*_KPC-51_, and *bla*_KPC-52_ genes have been reported in ST11-type K. pneumoniae (14, 15).

In this study, we report multiple variants of *bla*_KPC-2_ occurring in K. pneumoniae in two separated patients during their exposure to ceftazidime-avibactam for the treatment of infection and resulting in therapeutic failure. Among them, *bla*_KPC-76_ and *bla*_KPC-79_ were two novel *bla*_KPC_ alleles conferring resistance to ceftazidime-avibactam. These *de novo* mutations posed an unexpected challenge for both the diagnostic laboratory and clinical practice.

## RESULTS

### Case presentation.

**FIG 1:**
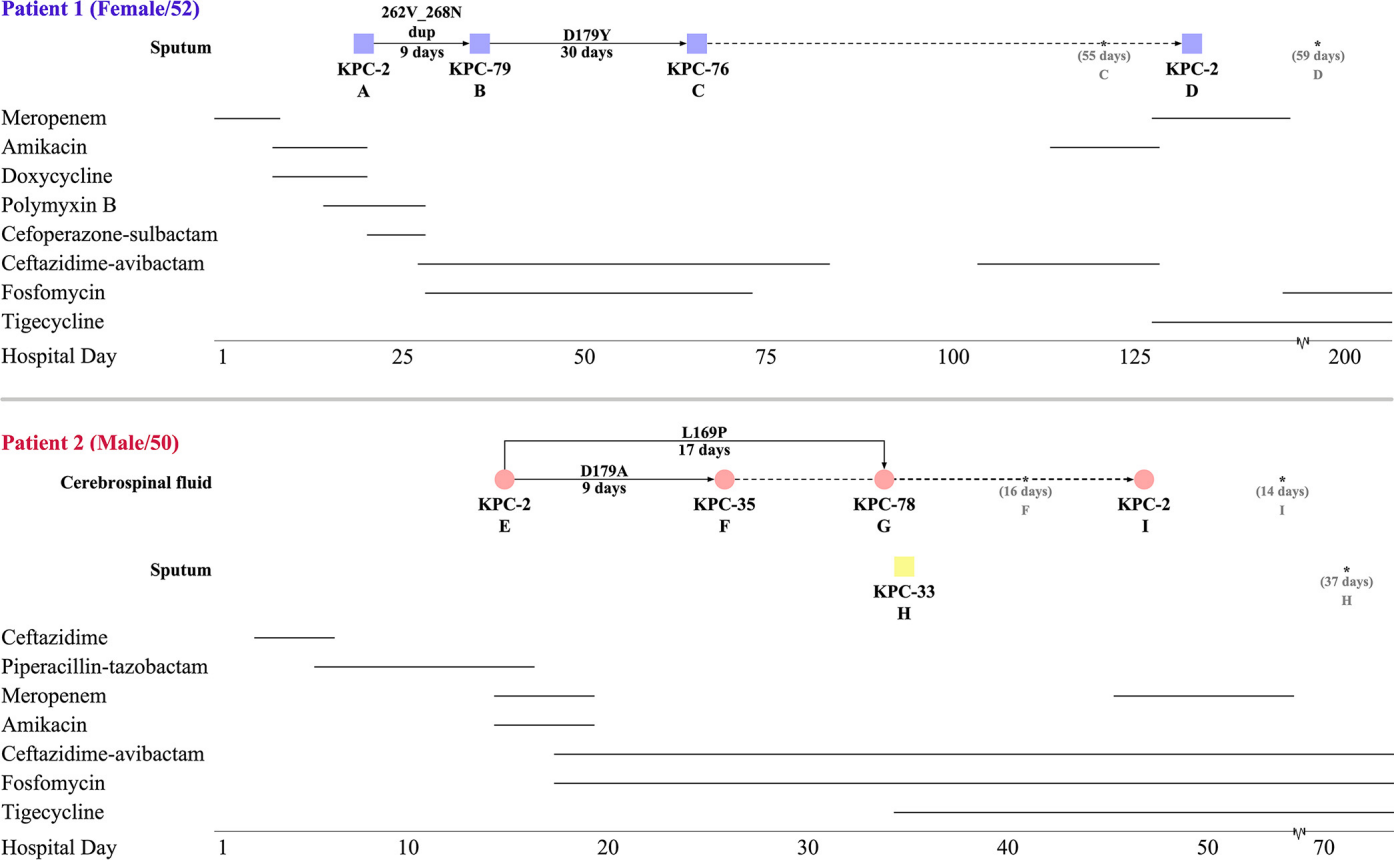
Clinical treatment and microbiological characteristics of patients infected with Klebsiella pneumoniae containing multiple variations in *bla*_KPC-2_. The colors indicate the strains isolated and their plasmids harboring *bla*_KPC_: the ST11 strain with plasmid pLWKPC (shown in purple), the ST859 strain with plasmid pZHKPC1 (red), and the ST11 strain with plasmid pZHKPC2 (yellow). Different geometries show different specimen sources. The last separation time of the same strain is asterisked and its existence time was also indicated. Solid arrows denote amino acid mutations between strains, while dotted arrows denote these potential ones, and the date below which presents the exposure duration of ceftazidime-avibactam before such mutation was found.

Two patients in this case shared similar infectious disease courses (Fig. 1). Originally, the carbapenem-resistant K. pneumoniae (CRKP) strains isolated were positive for *bla*_KPC-2_ gene, so ceftazidime-avibactam 2.5g q8h combined with fosfomycin and tigecycline (patient 2 only) replaced the empirical regimen. However, the poor prognosis was strongly associated with KPC-KP strain variability where multiple ceftazidime-avibactam-resistant K. pneumoniae carrying *bla*_KPC-2_ variants emerged during therapy. For patient 1, strain B (*bla*_KPC-79_ positive) was isolated only after 9-day ceftazidime-avibactam exposure, and strain C (*bla*_KPC-76_ positive) was isolated 30 days later. For patient 2, *bla*_KPC-35_-, *bla*_KPC-78_-, and *bla*_KPC-33_-positive K. pneumoniae (strain F, G, and H) were found after 9, 17, and 18 days of therapy, respectively. However, the *bla*_KPC-2_-positive strain D and I recurred as the dominant strains soon after meropenem was prescribed in the late stage of treatment. It is noteworthy that *bla*_KPC-33_-positive K. pneumoniae was isolated from the respiratory tract of patient 2 throughout the whole treatment, which wasn’t radically eliminated by either carbapenems or ceftazidime-avibactam.

### Basic characterization of K. pneumoniae strains.

**FIG 2:**
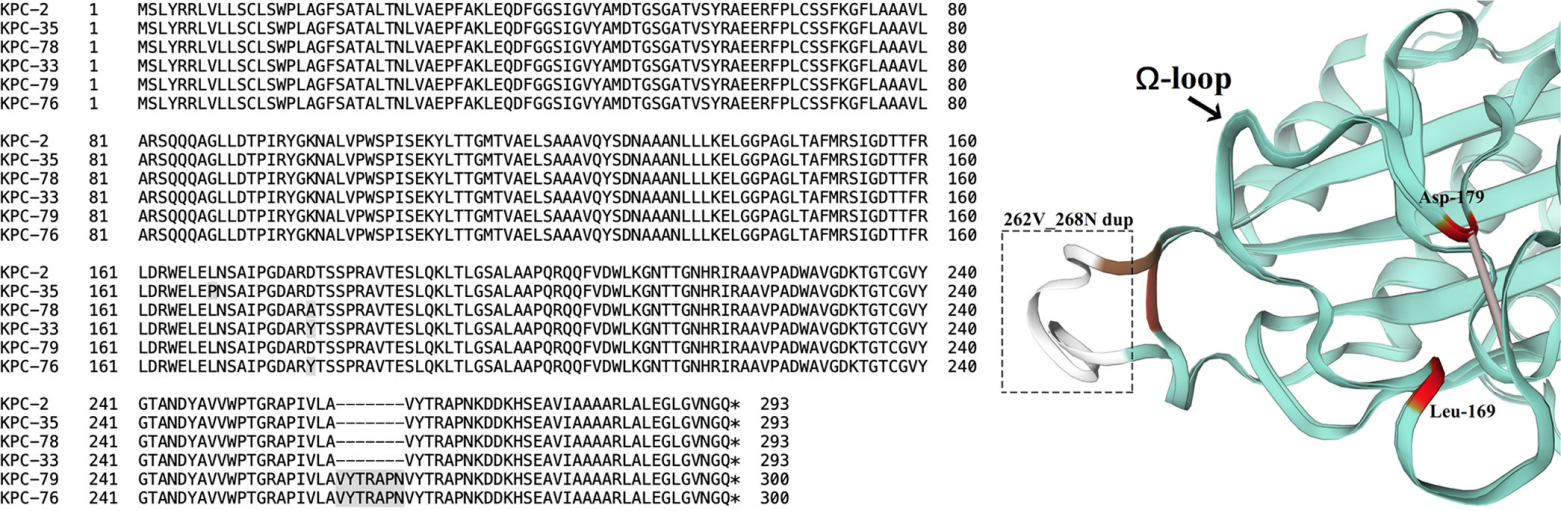
Amino acid sequence and X-ray crystallographic protein structure of KPC-2 and KPC-2 variants (SMTL ID: 6xd5.1). Mutation sites Leu169, Asp179, and amino acid tandem repeat (262V_269N dup) were highlighted and the Arg164 to Asp179 salt bridge of Ω-loop drawn as a short bar.

Per pulsed-field gel electrophoresis (PFGE), strains isolated from the same site of each patient were identical in genomic pulsotype and all were highly resistant to β-lactams, aminoglycoside, sulfanilamide, and quinolone antibiotics tested (Table 1, Fig. S1). A total of five *bla*_KPC-2_ variants were found, including *bla*_KPC-33_, *bla*_KPC-35_, and *bla*_KPC-78_ with mutation of Ω-loop from patient 2, and two novel alleles, *bla*_KPC-79_ and *bla*_KPC-76_, from patient 1. Therein, carbapenemase KPC-79 possessed a 7-amino-acid tandem repeat between amino acids 262 and 268 (262V_268N dup) compared with KPC-2 and further evolved into KPC-76 through the D179Y substitution during ceftazidime-avibactam exposure (Fig. 2). Compared with the *bla*_KPC-2_-positive K. pneumoniae, MICs of those with *bla*_KPC-2_ variants dropped from ≥64 mg/L to 0.25 to 8 mg/L for imipenem and meropenem and rose from 1 to 4 mg/L to ≥64 mg/L for ceftazidime-avibactam. Neither PAβN nor CCCP lowered their MIC for ceftazidime-avibactam. Only tigecycline, polymyxin B, meropenem-vaborbactam, and cefepime-zidebactam had good *in vitro* activity against all K. pneumoniae clinical isolates.

**TABLE 1:** MICs of K. pneumoniae strains isolated from patients, their *bla*_KPC_-positive E. coli DH5α transformants, and E. coli DH5α recipient strains

Isolations^*b*^	MIC (μg/mL)^*a*^																			
	IMP	MEM	ETP	CZA	MEV	FPT	FPZ	CZT	TGC	POL	SCF	TZP	FEP	CAZ	CRO	ATM	AMK	CIP	LEV	SXT
K. pneumoniae A (pLWKPC-KPC-2)	64	>64	>128	2	2	>64	2	>128	1	0.25	128	256	>128	>32	>32	>128	>128	>8	>16	>32
E. coli DH5α-A (pLWKPC-KPC-2)	2	2	2	0.25	≤0.015	1	0.125	4	0.125	0.5	32	256	4	>32	>32	>128	>128	≤0.06	≤0.125	≤0.25
K. pneumoniae B (pLWKPC-KPC-79)	4	8	32	64	2	64	2	>128	1	0.25	128	256	>128	>32	>32	>128	>128	>8	>16	>32
E. coli DH5α-B (pLWKPC-KPC-79)	0.5	≤0.03	≤0.06	4	≤0.015	0.125	0.125	16	0.25	0.25	16	8	4	>32	>32	64	>128	≤0.06	≤0.125	≤0.25
K. pneumoniae C (pLWKPC-KPC-76)	2	4	32	128	2	>64	4	>128	1	0.5	>128	>256	>128	>32	>32	>128	>128	>8	>16	>32
E. coli DH5α-C (pLWKPC-KPC-76)	0.25	≤0.03	≤0.06	16	≤0.015	0.06	0.125	8	0.25	0.25	16	4	4	>32	>32	64	>128	≤0.06	≤0.125	≤0.25
K. pneumoniae D (pLWKPC-KPC-2)	>64	>64	>128	2	2	>64	2	>128	1	0.25	128	256	>128	>32	>32	>128	>128	>8	>16	>32
K. pneumoniae E (pZHKPC1-KPC-2)	64	>64	>128	4	2	>64	2	>128	1	0.5	>128	>256	>128	>32	>32	>128	>128	>8	>16	>32
E. coli DH5α-E (pZHKPC1-KPC-2)	4	4	8	0.25	≤0.015	8	0.125	16	0.125	0.25	64	>256	8	>32	>32	>128	>128	≤0.06	≤0.125	≤0.25
K. pneumoniae F (pZHKPC1-KPC-35)	1	4	16	64	2	64	2	>128	1	0.25	>128	>256	128	>32	>32	128	>128	>8	>16	>32
E. coli DH5α-F (pZHKPC1-KPC-35)	0.125	≤0.03	≤0.06	4	≤0.015	0.125	0.125	64	0.125	0.25	32	16	4	>32	32	>128	>128	≤0.06	≤0.125	≤0.25
K. pneumoniae G (pZHKPC1-KPC-78)	0.5	2	32	128	1	64	2	>128	1	0.5	>128	>256	64	>32	>32	128	>128	>8	>16	>32
E. coli DH5α-G (pZHKPC1-KPC-78)	0.125	≤0.03	≤0.06	8	≤0.015	0.125	0.125	64	0.125	0.5	16	16	4	>32	32	>128	>128	≤0.06	≤0.125	≤0.25
K. pneumoniae H (pZHKPC2-KPC-33)	0.25	1	32	128	1	>64	0.5	>128	1	0.5	>128	>256	>64	>32	>32	>128	>128	>8	>16	>32
E. coli DH5α-H (pZHKPC2-KPC-33)	0.25	≤0.03	≤0.06	2	≤0.015	0.125	0.06	32	0.125	0.25	16	32	4	>32	>32	128	>128	≤0.06	≤0.125	≤0.25
K. pneumoniae I (pZHKPC1-KPC-2)	64	>64	>128	8	8	>64	4	>128	2	0.5	>128	>256	>128	>32	>32	>128	>128	>8	>16	>32
E. coli DH5α (-)	0.125	≤0.03	≤0.06	≤0.03	≤0.015	0.125	≤0.06	≤0.03	≤0.06	≤0.125	0.06	0.25	0.5	0.125	≤0.06	≤1	≤1	≤0.06	≤0.125	≤0.25
E. coli DH5α (pMD19-T-KPC-2)	16	8	8	0.5	≤0.015	32	0.25	128	0.25	0.25	>128	>256	64	>32	>32	>128	≤1	≤0.06	≤0.125	≤0.25
E. coli DH5α (pMD19-T-KPC-79)	2	0.5	0.5	64	≤0.015	8	0.125	>128	0.125	0.25	64	>256	16	>32	>32	64	≤1	≤0.06	≤0.125	≤0.25
E. coli DH5α (pMD19-T-KPC-76)	0.5	0.06	0.25	256	≤0.015	0.25	0.125	>128	0.25	0.25	32	32	8	>32	32	8	≤1	≤0.06	≤0.125	≤0.25
E. coli DH5α (pMD19-T-KPC-33)	0.25	0.06	0.25	64	≤0.015	0.25	0.25	>128	0.25	0.25	32	64	8	>32	32	8	≤1	≤0.06	≤0.125	≤0.25
E. coli DH5α (pMD19-T-KPC-35)	0.5	0.06	0.25	64	≤0.015	0.25	0.125	>128	0.25	0.25	32	32	8	>32	32	8	≤1	≤0.06	≤0.125	≤0.25
E. coli DH5α (pMD19-T-KPC-78)	0.5	0.06	0.25	128	≤0.015	0.25	0.125	>128	0.25	0.25	32	32	8	>32	32	16	≤1	≤0.06	≤0.125	≤0.25
E. coli DH5α (pMD19-T)	0.25	≤0.03	≤0.06	0.25	≤0.015	0.125	0.125	0.5	0.25	0.25	0.06	8	0.5	0.5	≤0.06	≤1	≤1	≤0.06	≤0.125	≤0.25

a IMP, imipenem; MEM, meropenem; ETP, ertapenem; CZA, ceftazidime-avibactam; CRO, ceftriaxone; CAZ, ceftazidime; FEP, cefepime; FPT, cefepime-tazobactam; FPZ, cefepime-zidebactam; CZT, ceftolozane-tazobactam; SCF, cefoperazone-sulbactam; TZP, piperacillin-tazobactam; ATM, aztreonam; AMK, amikacin; SXT, trimethoprim-sulfamethoxazole; CIP, ciprofloxacin; LEV, levofloxacin; TGC, tigecycline; POL, polymyxin B; MEV, meropenem-vaborbactam.

b K. pneumoniae clinical isolates were listed, followed closely by their transformants through electrotransformation, and the *bla*_KPC_ cloning strains were listed last. Information of plasmids carrying *bla*_KPC_ was given in parentheses behind each strain.

### Genotypic analysis of K. pneumoniae strains.

On the basis of the results of whole genome sequencing (WGS), K. pneumoniae isolated from the same site of each patient shared identities ranging from 99.972% to 99.993%. For patient 2, strain H isolated from the sputum only had a similarity of 80% in PFGE patterns with those from cerebrospinal fluid (CSF) (Fig. S1). Strains from the sputum (both patients) and CSF (patient 2) belonged to sequence type (ST) 11 and ST859, respectively. All isolates carried resistance genes including *rmtB*, *bla*_SHV-12_, *bla*_KPC_, *bla*_TEM-1B_, *bla*_LAP-2_, *fosA*, *sul2*, *dfrA14*, *catA2*, *qnrS1*, and *tet(A)*; meanwhile that from patient 1 also harbored *armA*, *aac(*3*)-IId*, *aadA2b*, *bla*_CTX-M-65_ (also in strain H), *bla*_DHA-1_, *sul1*, *qnrB4*, *oqxA*, *oqxB*, *mph(E)*, *mph(A)*, and *msr(E)* genes. Moreover, a premature stop codon occurred in the coding gene of *ompK35 by* the single nucleotide deletion at sequence position 86, and for OmpK36, glycine and aspartic acid were duplicated at amino acid position 134. Such changes for porins emerged in all K. pneumoniae isolated and might be another reason for the resistance of ceftazidime-avibactam.

### Location, function, and expression of *bla*_KPC_ genes.

**FIG 3:**
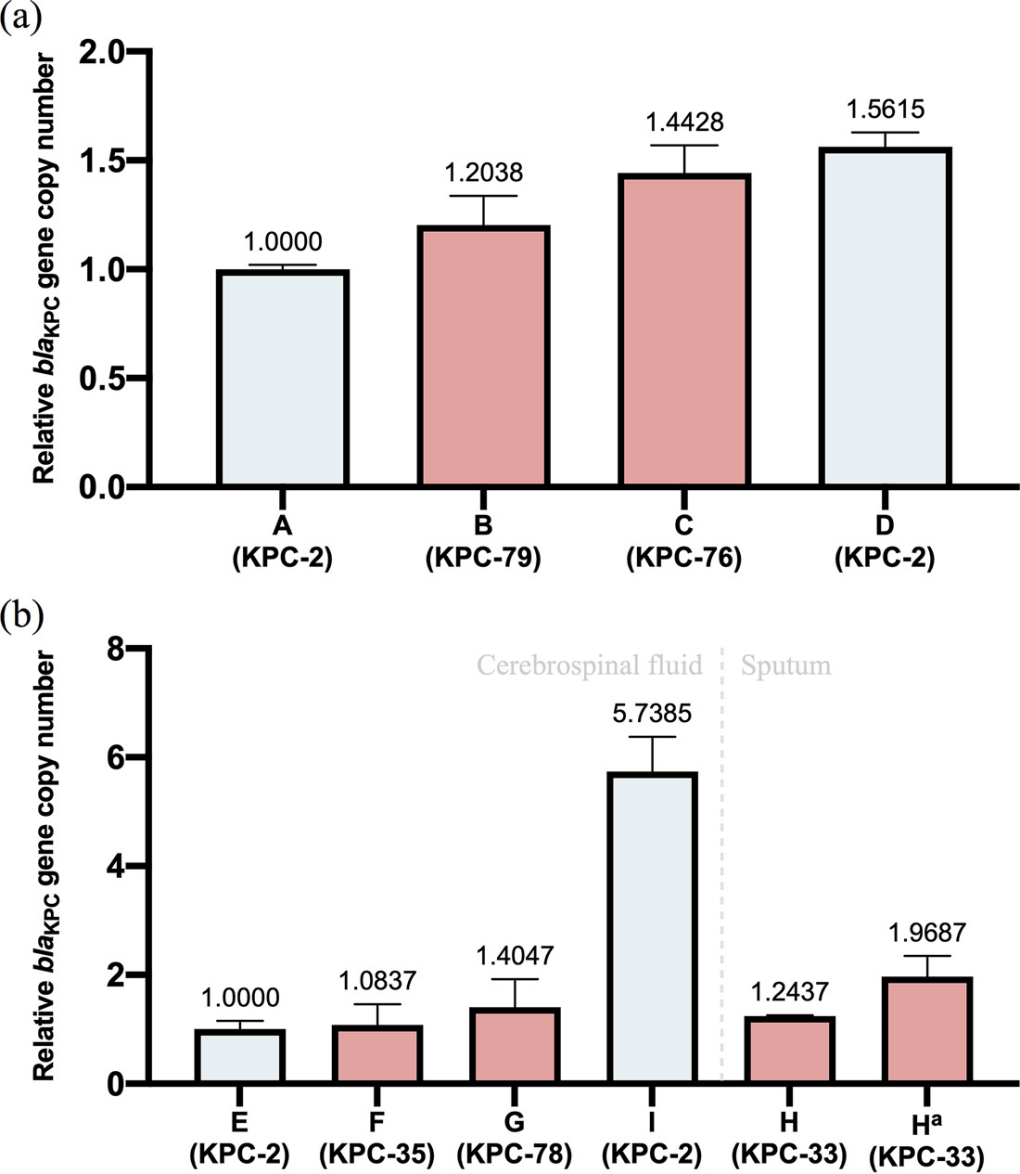
The expression level of *bla*_KPC_ genes in Klebsiella pneumoniae isolated during treatment. Picture (a) and (b) were relative copy numbers of strains (mean with 95% CI) from patient 1 and 2 comparing strain A and E, respectively, and those of *bla*_KPC-2-variant_ were highlighted. ^a^The relative copy number of *bla*_KPC-33_ gene in strain H during the late period of antimicrobial therapy.

The recombinant plasmids harboring *bla*_KPC_ were successfully transferred into recipient strain E. coli DH5α, altering their susceptibility to ceftazidime-avibactam, carbapenems, and cephalosporins (Table 1). In contrast with the *bla*_KPC-2_-positive clonal strain E. coli DH5α (pMD19-T-KPC-2), MICs of those with *bla*_KPC-2_ variants dropped from 16 to 8 mg/L to 0.06 to 2 mg/L to carbapenems and rose from 0.5 mg/L to ≥64 mg/L toward ceftazidime-avibactam. Resistance mediated by *bla*_KPC-79_ seemed in an intermediary state between *bla*_KPC-2_ and other variants, while *bla*_KPC-76_ led to a higher level resistance of ceftazidime-avibactam. In addition, transformants with *bla*_KPC_-positive plasmids extracted from K. pneumoniae clinical isolates obtained similar resistance of β-lactam antibiotics meanwhile acquired that of aminoglycosides (Table 1). Besides, the relative expression of the *bla*_KPC_ gene in strains isolated from the same patient gradually increase during treatment, especially in the late stage of infection. The copy number of *bla*_KPC_ gene in the last isolate of patient 1 could be 1.6-fold higher than the initial one, while that of patient 2 could even reach 5.7-fold (Fig. 3).

### Comparison of plasmids structures.

**FIG 4:**
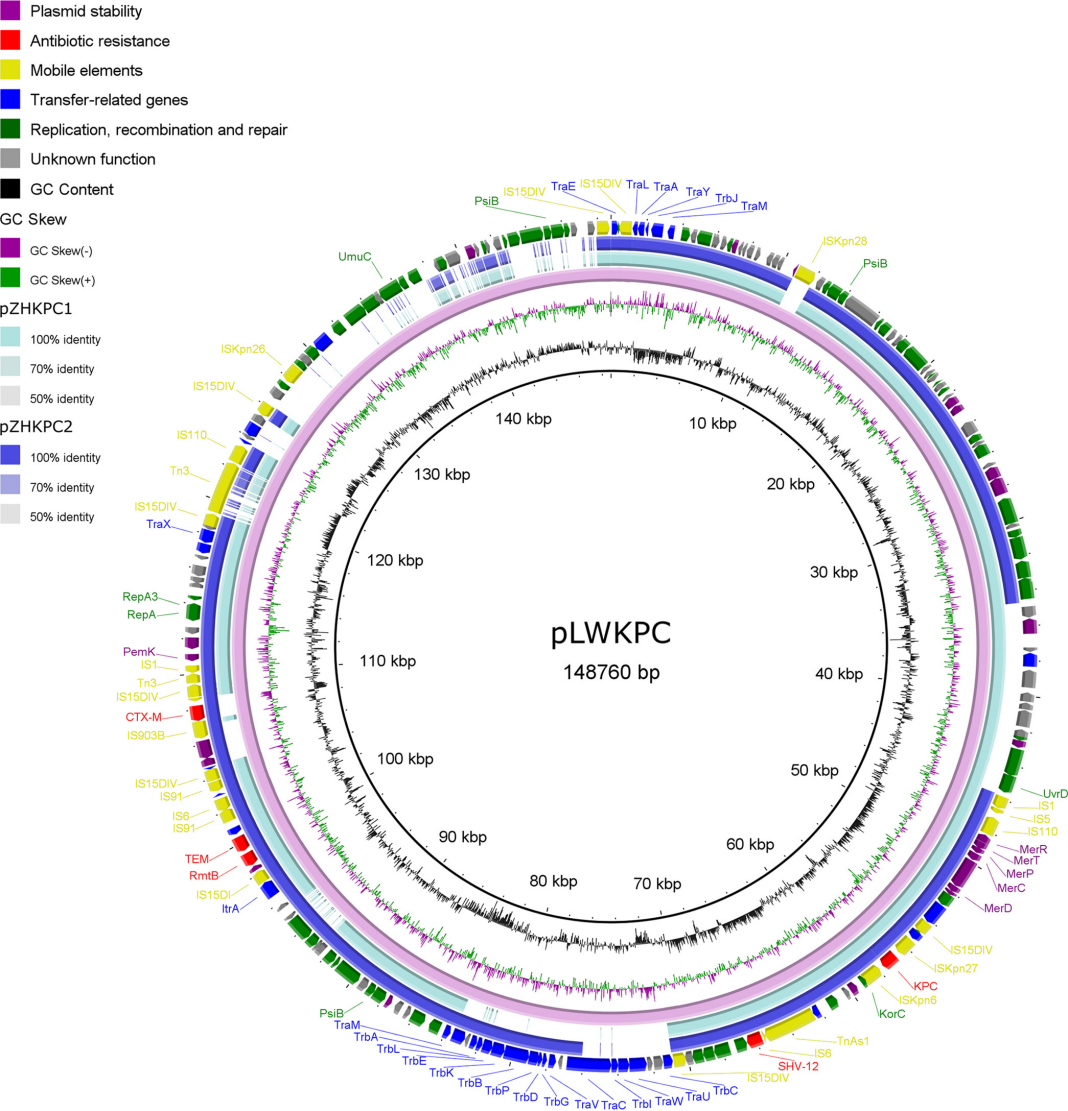
Comparative plasmid map of pLWKPC, pZHKPC1, and pZHKPC2. From the inside to the outside: circle 1, scale; circle 2, GC content; circle 3, GC Skew; circle 4, ring diagram of pLWKPC; circle 5, ring diagram of pZHKPC1; circle 6, ring diagram of pZHKPC2; circle 7, functional classified genes.

For K. pneumoniae A to D isolated from patient 1, *bla*_KPC_ was located in an IncFII/IncR plasmid pLWKPC, which was 148,760-bp in length and also carried *bla*_SHV-12_, *rmtB*, *bla*_CTX-M-65_, and *bla*_TEM-1B_ genes (Fig. 4). There were two multidrug resistance gene islands in plasmid pLWKPC, one contained *bla*_KPC_ and *bla*_SHV-12_, and the other included *rmtB*, *bla*_CTX-M-65_ and *bla*_TEM-1B_. Mobile genetic elements consisted of Tn3-family transposons, *IS15DIV*, *ISKpn6*, *ISKpn27*, *IS6*, *TnAs1*, etc., resulting in the potential quick transfer of antibiotic resistance genes. For K. pneumoniae isolated from patient 2, strain H had *bla*_KPC_ gene located on plasmid pZHKPC2 and other strains on pZHKPC1. Both plasmids were about 105-kb in length and resembled pLWKPC with 74% coverage and ≥99.88% identity (Fig. 4). Lacking the IncR replicon gene, plasmid pZHKPC2 belonged to the IncFII type. Plasmid pZHKPC1 and pZHKPC2 also contained two similar multidrug-resistance islands but with deletion of *bla*_CTX-M-65_ in the former one. Notably, plasmid pLWKPC perfectly matched with plasmid p44-2 (GenBank accession no. CP025463.1), showing the coverage and identity over 99%, with only deletion of small gene fragments. Plasmid p44-2 was found in K. pneumoniae isolated from the same hospital as this case, indicating the possibility of the horizontal transmission of plasmid like this.

In additional, large virulence plasmids were also found in K. pneumoniae strains from both patients. Virulence plasmid from strains of patient 1 was about 300 kb in length, containing a pathogenicity island with virulence gene siderophores aerobactin (*iucABCD*), hypermucoidy (*rmpA2*), and putative carboxymuconolactone decarboxylase family (*peg-589*), while that from strains of patient 2 contained an extra pathogenicity island with *rmpA* and *peg-344*, both pathogenicity islands that are common in China.

## DISCUSSION

Nowadays, KPC-KP related-infections are increasing rapidly worldwide (16). Ceftazidime-avibactam, as one of its most effective antibiotics, has already been clinically approved in the United States since 2015, in Europe since 2016, and in China since 2019, and its rising resistance is predictable with extensive use (17). Variation of *bla*_KPC_ gene emerged as an non-negligible mechanism of ceftazidime-avibactam resistance. To date, a total of 88 *bla*_KPC_ gene subtypes have been published, the detection of which has presented an uptrend since 2015 and has shown blowout growth since 2020, and 52.3% of them (46/88) were released after 2020. Based on previous reports and data from GenBank database (https://www.ncbi.nlm.nih.gov/pathogens/refgene/#gene_family:(blaKPC), by August 16, 2021), *bla*_KPC_ gene subtypes mediating inhibitor resistance have successively emerged by 2016 and are now mainly reported in Europe, China, and the United States (18–20). Especially in the recent 3 years, 53.8%(28/52) novel *bla*_KPC_ gene subtypes are resistant to inhibitors.

In China, KPC-2-producing strains associate with over half of CRE related-infections (21, 22), while other KPC carbapenemase alleles, including KPC-3 (23, 24), KPC-12 (25, 26), KPC-15 (27), KPC-16, and KPC-17 (28, 29), were sporadically reported in the last decade throughout the country. With the ever-growing use of ceftazidime-avibactam since 2019, inhibitor-resistant KPC-2 variants like KPC-33, KPC-51, KPC-52, and, KPC-74 began to be observed in clinically isolated ST11-type K. pneumoniae, among which only KPC-74 enzyme also mediated carbapenem resistance (14, 15, 30). Unlike in other countries, where ST258 K. pneumoniae led to the majority of KPC-related ceftazidime-avibactam resistant infections, the *bla*_KPC-2_-harboring K. pneumoniae isolated in our study belonged to ST859 and ST11, the former was rarely reported worldwide, while the latter might become the main type of ceftazidime-avibactam resistant strains in China (31).

Different from the previous clinical reports of a single variant, multiple different variants of a single *bla*_KPC-2_-positive strain were found in two patients this time, including mutations at different loci over the same period and successive variations to strengthen the resistance (32). A total of five kinds of *bla*_KPC_ alleles, including *bla*_KPC-33_, *bla*_KPC-35_, *bla*_KPC-76_, *bla*_KPC-78_, and *bla*_KPC-79_, were detected on IncFII/IncR-type plasmids that were also reported in *bla*_KPC-2_-positive strains from Shanghai and neighboring cities (33, 34). Such mutations firstly appeared after 9 to 18 days of medication and further evolved after another 30 days this time, which were typically reported to occur after 10 to 19 days, sometimes up to 33 days (31, 35).

All these *bla*_KPC-2_ variants conferred resistance to ceftazidime-avibactam and restored susceptibility to carbapenems. The mechanism for the KPC-79 enzyme might be that the β9-sheet and the following hinge to the α12-helix of KPC enzymes, which is proximal to the KPC-2 enzyme active site, is a variable region (36), KPC alleles like KPC-44 can have one- to 15-amino acid insertions in this region (37). On the flip side, the KPC-33, KPC-35, and KPC-78 enzymes presented with mutation within the Ω-loop, which enhanced ceftazidime affinity and reduced avibactam binding (38). The KPC-76 enzyme owned both kinds of mutations above. Because the resistance mediated by the KPC-79 enzyme shared an intermediary state between the KPC-2 enzyme and all other variants, D179Y mutation may be the main mechanism for drug resistance change for the KPC-76 enzyme, while amino acid insertion might be the secondary factor. The phenomenon mentioned above indicates selective pressure favoring variations of KPC-2 carbapenemase after short-term usage of ceftazidime-avibactam and its possibility of successive evolution during persistent exposure, which calls for urgent clinical attention.

Problems arose in the clinical detection of these novel KPC variants owing to their inconspicuous resistant characteristic of carbapenems and lack of routine ceftazidime-avibactam susceptibility testing. Routine detection methods of carbapenemase-like modified carbapenem inactivation method (mCIM)/EDTA-modified CIM (eCIM) recommended by CLSI and 3-aminophenylboronic acid (APB) or EDTA synergy test show negative results for these isolates, whereas the detection results of GeneXpert Carba-R are all positive, suggesting an indeterminate KPC allele, which is often speculated as KPC-2 type in China (39–41). All test results might lead to incorrect prediction of ceftazidime-avibactam susceptibility, resulting in incorrect clinical usage. Therefore, it is urgent to explore a feasible method for accurate detection of different types of KPC enzyme.

Unfortunately, even with an accurate diagnosis, K. pneumoniae-producing KPC variants often led to poor prognosis despite its reduced resistance of carbapenems (13). Firstly, the *bla*_KPC-2_-containing K. pneumoniae dominated again soon after the carbapenems substitution therapy for ceftazidime-avibactam-resistant strains (14). Moreover, when the therapeutic regimen was adjusted into ceftazidime-avibactam combined with carbapenems, the increased expression of *bla*_KPC-2_ gene and point mutation of OmpK35 and OmpK36 mediated drug resistance and prevented successful treatment (10, 42, 43). Finally, with combination medication, the K. pneumoniae with *bla*_KPC-33_ could not be eliminated in patient 2. On one hand, the clinical success of ceftazidime-avibactam treatment among patients with pneumonia was much lower than those with other CRE-related infections (44). The possible reason might be that the concentration of avibactam in the epithelial lining fluid(ELF) was below the concentration needed to suppress resistance amplification of KPC-KP (45, 46). In our study, low concentration and possible limited CSF penetration of avibactam could further lead to a predisposition for *bla*_KPC_ gene mutation. On the other hand, the expression level of *bla*_KPC-33_ gene also increased in the late stage of treatment. Such strain seemed to translocate to the respiratory tract instead of evolving independently, because it wasn’t related to those from cerebrospinal fluid and no *bla*_KPC-2_-positive strain was isolated during the whole treatment process. Mercifully, although strains carried a large virulent plasmid, one nucleotide deletion at the poly(G) tract in the *rmpA2* gene might lead to the reduction of its virulence (47).

Novel KPC variants have constantly appeared worldwide in recent years, for bacteria mutated to survive from the powerful selective pressure by antibiotics, which might be related to the use of new enzyme inhibitors, like avibactam, vaborbactam, and relebactam. Worryingly, the appearance of novel KPC variants could rise steadily followed by the increase of clinical application of such drugs in the treatment of KPC-KP-related infections. Therefore it is necessary for medical staff to monitor changes in the drug resistance and confirm carbapenemase type in special cases to guide the anti-infection treatment for CRE infection accurately.

## MATERIALS AND METHODS

### Clinical isolates and patients’ data.

Two inpatients were found infected with ceftazidime-avibactam-resistant K. pneumoniae due to multiple *bla*_KPC-2_ mutants in Huashan hospital in Shanghai. Patient 1, a 52-year-old woman, was admitted in neurology for autoimmune encephalitis and pneumonia in August 2019. Patient 2, a man aged 50, was hospitalized for diffuse large B-cell lymphoma in July 2020 and developed disturbance of consciousness and progressive pulmonary infection soon after stereotactic biopsy. A total of four (strains A to D) and five (strains E to I) K. pneumoniae strains were isolated during the hospitalization of patient 1 and 2, respectively. Carbapenem-resistant Klebsiella pneumoniae (CRKP) strain A and E were initially isolated from sputum and cerebrospinal fluid. Still, along with the clinical usage of ceftazidime-avibactam, strains B, C, F, and G from the above sites were confirmed ceftazidime-avibactam resistant with a MIC of ≥64 mg/L, while its MICs of carbapenems dropped greatly. For patient 2, the similar K. pneumoniae strain H was also found in sputum. However, the CRKP strain D and I were isolated when the regimen was switched to meropenem. All strains were identified by MALDI-TOF MS (bioMérieux, Marcy-l’Étoile, France).

### Antimicrobial susceptibility testing.

MICs were determined with a broth microdilution method according to the Clinical and Laboratory Standards Institute (CLSI) (48). Antimicrobial agents such as β-lactam, β-lactam/β-lactamases inhibitor combinations (including ceftazidime-avibactam and meropenem-vaborbactam), sulfonamides, amikacin, tigecycline, and polymyxin B were tested, with quality control and interpretation of the results according to 2021 CLSI breakpoints for all agents with the exception of tigecycline and polymyxin (48). The MICs of tigecycline and polymyxin were interpreted using the MIC breakpoints for *Enterobacterales* of U.S. FDA and EUCAST, respectively (49, 50). As efflux pump inhibitors, 25 mg/L phenyl-arginine-β-naphthylamide (PAβN) and carbonyl-cyanide-chlorobenzene-hydrazine (CCCP) were added respectively into broth to observe the MIC variation of ceftazidime-avibactam, and a MIC decrease for no less than quadruple was considered to be significant.

### Pulsed-field gel electrophoresis.

With Salmonella *braenderup* H9812 as the reference marker, bacterial DNA was digested with *Xba I* and S1-nuclease and subjected to PFGE and S1-PFGE, respectively. PFGE was carried out using a CHEF Mapper system (Bio-Rad Laboratories, Hercules, CA, USA). According to the criteria of Tenover et al. (51), PFGE patterns were interpreted using BioNumerics software version 6.5 to analyze the genetic relationship among K. pneumoniae strains.

### *bla*_KPC_ cloning and plasmid transformation experiments.

Plasmid DNA was extracted by phenol-chloroform from donors harboring *bla*_KPC-2_ or its mutant and were transferred to Escherichia coli DH5α by electroporation. Meanwhile, *bla*_KPC_, including putative promoter, were amplified from plasmid DNA by PCR using primers KPC-F-KpnI (5′-CGGGGTACCGGTCGTATCAGCGACATCGT-3′) and KPC-R-XbaI (5′-CCGTCTAGATCTGAGGCGAATGGCGTATC-3′). The PCR product (1.6-kp) was then purified using a QIAquick PCR purification kit (Qiagen), and cloned into the pMD 19-T-vector (TaKaRa, Dalian, China) before transformed into E. coli DH5α by chemical transformation. These transformants were selected on Luria-Bertani agar plates containing ampicillin (50 mg/L) and were verified by PCR for the presence of *bla*_KPC_, whose positive products were then sequenced and blasted (http://blast.ncbi.nlm.nih.gov/BLAST.cgi).

### Quantitative real-time PCR.

All K. pneumoniae strains were examined by qualitative real-time PCR (qRT-PCR) to assess the *bla*_KPC_ expression normalized to an internal K. pneumoniae housekeeping gene *rrsE*, as previously described (52). RNA was obtained from late-exponential-phase cultures and reverse transcripted to cDNA (MiniBEST Universal RNA Extraction Kit; PrimeScrip RT reagent kit with gDNA Eraser, TaKaRa, Dalian, China). qRT-PCR was then performed by the Applied Biosystems Viia 7 real-time PCR system, with *bla*_KPC_ primers KPC-F (5′- GGCCGCCGTGCAATAC-3′) and KPC-R (5′-GCCGCCCAACTCCTTCA-3′) and the SYBR Premix *Ex Ta* (TaKaRa, Dalian, China).

### Whole-genome sequencing and analysis.

Total DNA of K. pneumoniae strains and transformants was extracted and subjected to WGS on a Nova-seq platform (Illumina, San Diego, CA, USA) short-read sequencing as 150-bp paired-end reads, and then *de novo* assembled by SPAdes 3.12.0 (53). Multi-locus sequence typing (MLST) and antimicrobial resistance genes analysis were identified using MLST 2.0 (https://cge.food.dtu.dk/services/MLST/), and ResFinder 4.1 (https://cge.food.dtu.dk/services/ResFinder/), respectively.

### Data availability

The nucleotide sequence of pZHKPC1, pZHKPC2, and pLWKPC harboring *bla*_KPC-79_ and the fragment with *bla*_KPC-76_ were deposited into the GenBank under accession numbers OM928502, OM928503, MT875328, and MT550690, respectively.
