# Supplementary material for: Multiple Novel Ceftazidime-Avibactam-Resistant Variants of blaKPC-2-Positive Klebsiella pneumoniae in Two Patients
Source: Microbiol Spectr. 2022 May 19;10(3):e01714-21. doi: 10.1128/spectrum.01714-21 (PMC9241591; doi:10.1128/spectrum.01714-21)
Supplement: SUPPLEMENTAL FILE 1 — Supplemental material. Download spectrum.01714-21-s001.pdf, PDF file, 1.1 MB [file spectrum.01714-21-s001.pdf]

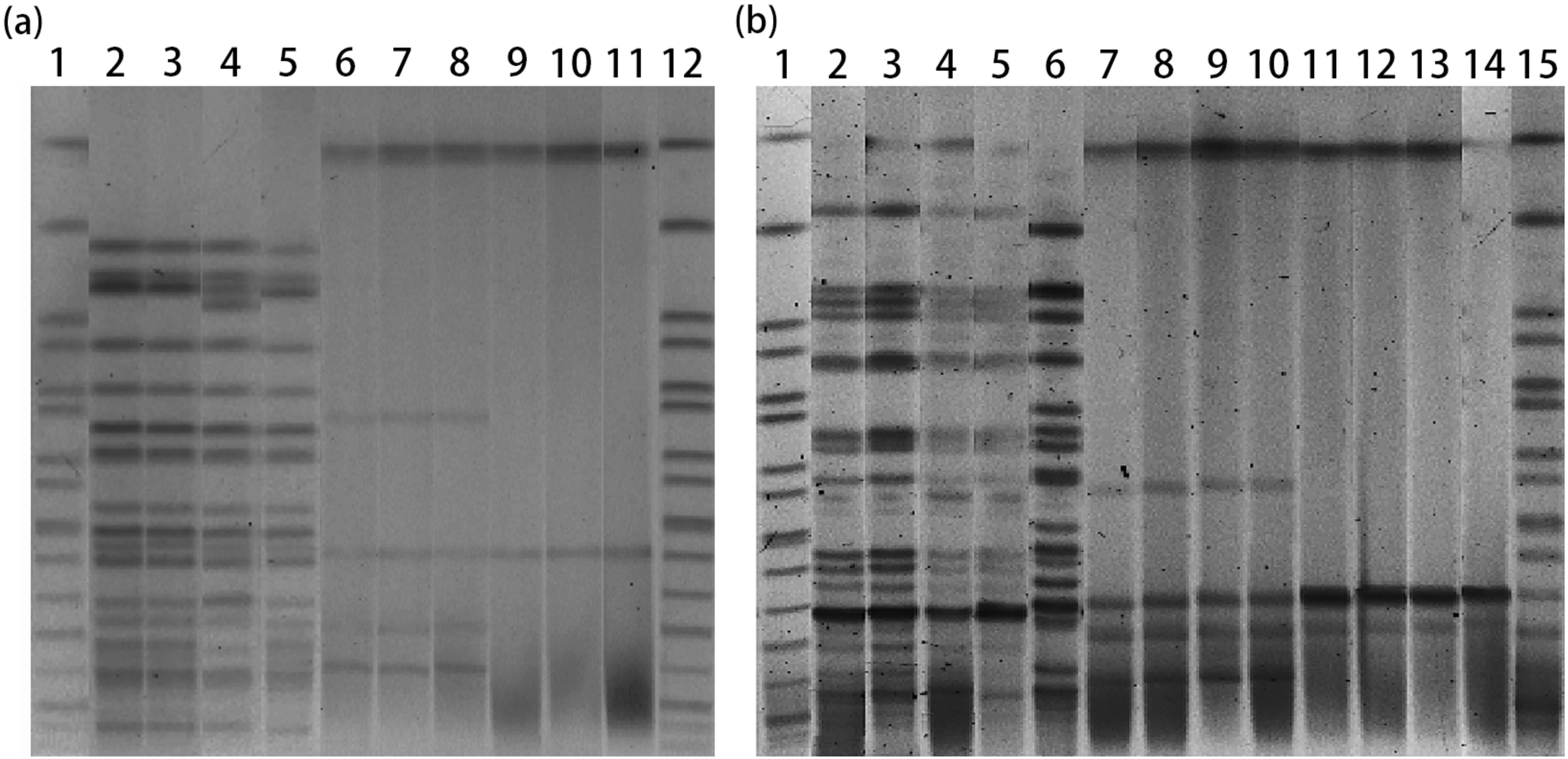

Figure S1. PFGE and S1-PFGE of *Klebsiella pneumoniae* clinical isolates and *bla*$_{KPC}$-positive transformants.

Picture a: Lanes 1 and 12, marker Salmonella braenderup H9812; line 2 to 5, PFGE image of *K. pneumoniae* A to D; line 6 to 11, S1-PFGE image of *K. pneumoniae* A to C and *E. coli* DH5α-A to -C. Picture b: Lanes 1 and 15, marker Salmonella braenderup H9812; line 2 to 6, PFGE image of *K. pneumoniae* E, F, G, I, and H; line 7 to 14, S1-PFGE image of *K. pneumoniae* E to H and *E. coli* DH5α-E to -H.
